# Safety and Efficacy of Simplified EMR Versus ESD for Rectal Neuroendocrine Tumors ≤ 10 Mm: A Retrospective Cohort Study

**DOI:** 10.3390/jcm14176125

**Published:** 2025-08-29

**Authors:** Linfeng Zou, Long Zou, Yingyun Yang, Weixun Zhou, Xi Wu, Tao Guo, Qingwei Jiang, Yunlu Feng, Shengyu Zhang, Qiang Wang, Aiming Yang

**Affiliations:** 1 Department of Gastroenterology, Peking Union Medical College Hospital, Peking Union Medical College and Chinese Academy of Medical Sciences, Beijing 100730, China; zoulf11@163.com (L.Z.);; 2 Department of Pathology, Peking Union Medical College Hospital, Peking Union Medical College and Chinese Academy of Medical Sciences, Beijing 100730, China

**Keywords:** rectal neuroendocrine tumor, endoscopic mucosal resection, endoscopic submucosal dissection, R0 resection, cost-effective

## Abstract

**Background:** Rectal neuroendocrine tumors (NETs) ≤ 10 mm are commonly managed by endoscopic resection. However, the optimal technique remains controversial. We aimed to compare the efficacy and safety of a simplified endoscopic mucosal resection (sEMR) technique, performed without submucosal injection, with conventional endoscopic submucosal dissection (ESD) for small rectal NETs. **Methods:** This retrospective, single-center study included 74 patients with histologically confirmed rectal NETs ≤ 10 mm treated with sEMR (*n* = 37) or ESD (*n* = 37) between January 2022 and January 2025. Patients in the ESD group were matched 1:1 by age and gender. Baseline characteristics, procedural outcomes, histopathologic findings, and cost were analyzed. The primary outcome was histological complete resection (R0) rate; secondary outcomes included en bloc resection, intraoperative bleeding, tumor-to-margin distance, operation time, and material costs. **Results:** The R0 resection rate was significantly higher in the sEMR group compared to the ESD group (91.9% vs. 67.6%; *p* = 0.019). Tumor-to-margin distance was also significantly greater in the sEMR group [median (IQR): 0.2 (0.1–0.3) mm vs. 0.1 (0–0.2) mm; *p* = 0.024]. Intraoperative bleeding was less frequent in the sEMR group (2.7% vs. 21.6%; *p* = 0.028). Median operation time (409 vs. 1469 s; *p* < 0.001) and material cost (1486 vs. 6390 CNY; *p* < 0.001) were both significantly lower in the sEMR group. **Conclusions:** Compared with ESD, the simplified EMR technique without submucosal injection demonstrated higher R0 resection rates, lower bleeding risk, shorter operation time, and lower costs for small rectal NETs. sEMR may offer a safe, efficient, and cost-effective alternative in selected patients.

## 1. Introduction

Rectal neuroendocrine tumors (NETs) are subepithelial neoplasms with intact surface mucosa extending from neuroendocrine cells in epithelial crypts into lamina propria (mucosae) or submucosa [1]. They are rare but increasingly diagnosed with screening colonoscopy [2]. They are pathologically categorized according to the World Health Organization classification system, and their pathological grade is based on the mitotic and Ki-67 indices [3]. Although rectal NETs can range in size, those measuring ≤ 10 mm are often confined to submucosa, typically presenting as small, sessile lesions with a relatively low risk of metastasis [4], and the long-term survival probability of patients without metastasis at the time of diagnosis is 100% [5].

National Comprehensive Cancer Network (NCCN) guidelines recommend that for small (<1 cm) incidental lesions, complete endoscopic resection with negative margins may be sufficient [6]. However, incomplete resection of rectal NETs poses a significant clinical challenge, with the incomplete resection rate ranging from 10% to 60% [7,8,9,10]. The optimal techniques remain a matter of debate. Many endoscopic methods for the management of small NET have been reported, including simple polypectomy, endoscopic mucosal resection (EMR), EMR with a ligation device (EMR-L) [11,12], EMR using a transparent cap (EMR-C) [13], precut EMR (EMR-P) [14], ligation-assisted EMR (ESMR-L) [15], anchored snare-tip EMR (ASEMR) [16], and endoscopic submucosal dissection (ESD) [17]. Among these, simple polypectomy is the least invasive but also the least effective, with reported histological complete resection (R0) rates as low as 20–30% [18,19]. Nearly all other procedures involve submucosal injection of a saline solution beneath the tumor to elevate the lesion and reduce the risk of perforation or positive deep resection margins. Recently, a newly proposed modified cap-assisted EMR procedure omitted submucosal injection in certain patients, with a similar complete resection rate [20]. Another study compared ESMR-L with and without submucosal injection found that ESMR-L without injection achieved significantly greater tumor-free vertical margin distances and a higher sufficient vertical R0 resection rate without increasing adverse events [21]. These updates reflect a growing trend towards minimizing procedural complexity while maintaining high resection quality and safety.

In our endoscopy center, we employed a simplified EMR technique omitting submucosal injection for the resection of rectal NETs in certain patients. Our study aims to further evaluate the efficacy and safety of sEMR and ESD for the treatment of rectal NETs, focusing on complete resection rates and complications. By analyzing data from a well-controlled cohort of patients who underwent ESD, we seek to provide evidence that the simplified procedure could also be utilized to manage rectal NET while maintaining high resection quality and safety.

## 2. Method

### 2.1. Study Design

This study is a single-center, retrospective analysis conducted at the Endoscopy Center of Peking Union Medical College Hospital, a tertiary academic referral institution. The study was designed to evaluate the efficacy and safety of simplified endoscopic mucosal resection (sEMR) compared to conventional endoscopic submucosal dissection (ESD) in treating rectal neuroendocrine tumors (NETs) ≤10 mm in size. The study was conducted in accordance with the Declaration of Helsinki and approved by the Ethics Committee of Peking Union Medical College Hospital (approval No.I-23PJ924 and 14 June 2023). To cover the additional analysis of R1 resection rates identified during the study, an updated approval was obtained (approval No. I-25PJ1564 and 15 July 2025). Written informed consent for ESD or sEMR was obtained from all patients before the procedure. The need for additional written informed consent specifically for this study was waived due to the general consent for retrospective studies on participating hospital in-patient consent forms.

### 2.2. Patient Population

This study included all patients who underwent sEMR and matched patients who underwent ESD for rectal neuroendocrine tumors (NETs) between from 1 January 2022 to 30 June 2025. Patients who underwent ESD were selected as a control group using 1:1 matching based on age (±5 years) and sex. The lesions were initially identified as submucosal tumors during colonoscopy, with or without endoscopic ultrasound. The decision to perform sEMR or ESD was made by the endoscopists based on the lesion’s characteristics and the patient’s suitability for the procedure. Only patients with a histologically confirmed NET with a diameter of no more than 10 mm were included. Exclusion criteria included prior surgical resection of the same lesion and presence of synchronous advanced malignancies.

### 2.3. Interventions

All endoscopic procedures were performed by experienced therapeutic endoscopists (each with >500 ESD/EMR procedures performed) using high-definition colonoscopes. In the majority of cases, endoscopic ultrasonography (EUS) was employed to aid in treatment planning.

The decision to perform sEMR or ESD was made based on clinical judgment by the attending endoscopist. Factors considered in the decision-making process included lesion size, location, morphology, accessibility, endoscopic ultrasound (EUS) findings, and the operator’s assessment of technical feasibility.

#### 2.3.1. Procedure of sEMR

**Figure 1:**
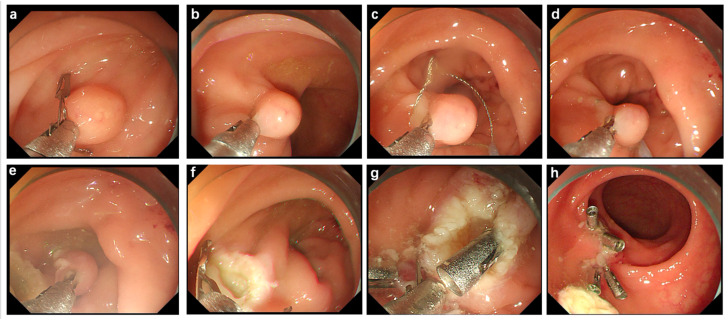
Process of simplified EMR. (**a**,**b**) The lesion is localized and elevated with the aid of a titanium clip placed at its base. (**c**,**d**) A snare is employed to securely capture the lesion. (**e**,**f**) The captured lesion is resected using electrocautery. (**g**,**h**) The resection surface is densely closed with titanium clips to prevent postoperative bleeding.

The sEMR procedure was performed according to the stepwise approach illustrated in Figure 1. A forward-viewing colonoscope equipped with a transparent distal attachment cap. Before insertion, an electrosurgical snare was pre-mounted externally around the cap. The snare loop was pre-expanded and fixed in the open position before insertion, ensuring readiness for immediate lesion capture without further device manipulation. The endoscope was advanced into the rectum with the preloaded snare. The lesion was identified endoscopically as a submucosal tumor, based on its morphological features and submucosal elevation. A single endoscopic clip was applied to the base of the lesion to lift the lesion, which provided mechanical traction, facilitating upward lifting of the lesion to optimize snare capture. The preloaded snare was then advanced and positioned over the elevated lesion, encircling it precisely. With the lesion securely captured, electrosurgical cautery was applied to perform complete resection in a controlled and en bloc fashion. The post-resection wound bed was carefully inspected. Visible vessels at the resection site were clipped to prevent delayed bleeding. A repeat endoscopic inspection was performed to confirm complete lesion removal and to check for any signs of residual tissue, perforation, or active bleeding. During the process, no submucosal injection was performed.

#### 2.3.2. Procedure of ESD

Endoscopic submucosal dissection (ESD) was performed following a standardized protocol to achieve en bloc resection of the lesion. The procedure began with clear endoscopic identification of the lesion, followed by the creation of marking dots circumferentially around its margins using a coagulation current. After marking, a submucosal injection was administered using a solution of diluted saline mixed with methylene blue, which served to elevate the lesion from the muscularis propria and enhance visualization of tissue planes. A circumferential mucosal incision was then made outside the marked area using an endoscopic knife, creating an access point for subsequent dissection. Submucosal dissection was performed meticulously with the aid of electrosurgical tools, allowing for gradual and controlled separation of the submucosal layer from the underlying muscle, thereby achieving complete en bloc removal of the lesion. The resected specimen was carefully retrieved and immediately prepared for histopathological examination. To prevent delayed bleeding, all visible vessels exposed on the resection bed were coagulated or clipped using hemostatic forceps or endoscopic clips as appropriate. Throughout the procedure, careful attention was paid to maintain clear visualization and avoid inadvertent perforation or thermal injury to the muscularis propria.

### 2.4. Pathological Evaluation

All resected specimens were immediately fixed in 10% neutral-buffered formalin and subsequently embedded in paraffin. Serial sections were cut at 2–3 mm intervals perpendicular to the mucosal surface and stained with hematoxylin and eosin (H&E) for routine histological evaluation. Tumor size and histological type were assessed by experienced gastrointestinal pathologists according to the 2019 WHO classification of digestive system tumors [22]. Tumor grade was recorded as G1 or G2 based on mitotic count and Ki-67 proliferation index.

The status of horizontal and vertical resection margins was determined, with margins considered positive if tumor cells were present at the resection line. Lymphovascular invasion was also evaluated. Immunohistochemical staining for Chromogranin A (CgA) and Synaptophysin (Syn) was performed to confirm neuroendocrine differentiation and progression. Additionally, the shortest distance from the tumor edge to the nearest resection margin (tumor-to-margin distance) was measured using digital slide analysis software when applicable. All pathological evaluations were independently verified by at least two pathologists in a blinded manner.

### 2.5. Data Collection

Clinical data were retrospectively collected from patient medical records, including demographic, clinical, endoscopic, histopathological, and procedural information. Demographic characteristics included patient age and gender. Clinical presentation was categorized according to the indication for endoscopic examination, such as incidental findings, abdominal pain or distension, altered bowel habits, or other related gastrointestinal symptoms.

Endoscopic variables included the anatomical location of the tumor, maximal diameter, intraoperative events (such as bleeding or perforation), and whether en bloc resection was achieved. Histopathological data included tumor grade (G1 or G2), status of horizontal and vertical resection margins (positive or negative), presence of lymphovascular invasion, and immunohistochemical staining results for Chromogranin A (CgA) and Synaptophysin (Syn). Additionally, the shortest distance from the tumor edge to the resection margin (tumor-to-margin distance) was measured on histological slides by pathologists, and this metric was compared between the sEMR and ESD groups. Professional fees, including operator labor costs, anesthesia fees, and other personnel-related charges, were not included in the cost analysis.

Procedural parameters included operation time, which was defined as the time interval from the first endoscopic photograph taken before resection to the final photograph taken at the end of the procedure. Material cost was calculated based on itemized hospital billing records, covering consumables and endoscopic devices used during the procedure.

### 2.6. Outcome Measures

The primary outcome was the histological complete resection rate, defined as en bloc resection of the tumor with histologically confirmed negative horizontal and vertical margins (R0 resection). Secondary outcomes included (1) en bloc resection rate (regardless of margin status), (2) intraoperative or delayed bleeding rate, (3) tumor-to-margin distance, as measured microscopically on histological sections, (4) operation time, defined as the duration between the first and last endoscopic images during the procedure, and (5) material cost, based on the total expense of consumables and endoscopic instruments used, excluding operator-related fees.

### 2.7. Statistical Analysis

Statistical analysis was performed using R 4.5.1. Continuous variables were expressed as mean ± standard deviation (SD) or median (range) depending on their distribution. Parametric tests, such as the Student’s *t*-test, were used for comparisons of normally distributed variables. For non-normally distributed variables, non-parametric tests, such as the Mann–Whitney U test, were employed. Categorical variables were presented as counts and percentages and were compared using the chi-square test or Fisher’s exact test. Multivariate analysis was performed using Firth’s penalized logistic regression to account for small sample size and rare events. Results were reported as odds ratios (ORs) with 95% confidence intervals (CIs) and *p*-values. The level of significance was set at a two-tailed *p*-value of < 0.05.

## 3. Result

### 3.1. Baseline Characteristics

A total of 74 patients with histologically confirmed rectal neuroendocrine tumors (NETs) were included, with 37 patients each in the sEMR and ESD groups. Age and gender were well matched between groups. As shown in Table 1, the mean age was 49.4 ± 11.0 years in the sEMR group and 48.6 ± 10.3 years in the ESD group (*p* = 0.752). The male-to-female ratio was identical in both groups (16:21; *p* = 1.000).

Most tumors were incidentally discovered during screening colonoscopy (67.6% in sEMR vs. 75.7% in ESD). Other presenting symptoms, such as abdominal pain/distention (16.2% vs. 10.8%) and altered bowel habits (13.5% vs. 10.8%), were similarly distributed (*p* = 0.878).

No significant differences were found between the two groups in tumor size [median 5 mm (IQR: 5–7) vs. 6 mm (4–8); *p* = 0.804], distance from the anal verge [5 cm (5–8) vs. 5 cm (4–8); *p* = 0.611], or the use of preoperative endoscopic ultrasound (83.8% vs. 75.7%; *p* = 0.563). All patients achieved en bloc resection. However, intraoperative bleeding occurred significantly more often in the ESD group (21.6%) than in the sEMR group (2.7%; *p* = 0.028). No perforations were reported in either group.

### 3.2. Operation Time and Material Cost

The operation time and material costs were significantly reduced in the sEMR group compared with the ESD group. Specifically, the median operative time in the ESD group was 1469 s (IQR: 1106–1803), whereas in the sEMR group it was 409 s (IQR: 324–648) (*p* < 0.00001). Furthermore, the median material cost in the ESD group was 6390 CNY (IQR: 5159–7713), which was significantly higher than that in the new group (1486 CNY [IQR: 1341–1565], *p* < 0.001).

### 3.3. Histopathological Findings

As summarized in Table 2, the R0 resection rate was significantly higher in the sEMR group (91.9%) compared to the ESD group (67.6%; *p* = 0.019), indicating a better histological clearance with sEMR.

There were no significant differences between the two groups in tumor grade (*p* = 0.560) or in the presence of lymphovascular invasion (2.7% vs. 5.4%; *p* = 1.000). Regarding immunohistochemical findings, Chromogranin A (CgA) positivity was observed in 64.7% of sEMR cases and 66.7% of ESD cases (*p* = 1.000), while Synaptophysin (Syn) positivity was nearly universal in both groups (100% vs. 97.2%; *p* = 1.000).

### 3.4. Margin Status Analysis

Beyond the type of procedure, other factors potentially associated with R1 resection were investigated by stratifying patients into R0 (*n* = 59) and R1 margin (*n* = 15) groups (Table 3). No significant differences were observed between the groups in terms of age, sex, presenting symptoms, tumor size, tumor location (distance from anal verge), tumor grade, or preoperative endoscopic ultrasound use (*p* > 0.05 for all).

Notably, lymphovascular invasion was exclusively observed in the R1 resection group (20.0% vs. 0%; *p* = 0.006), suggesting a potential link with incomplete resection. Although not statistically significant, CgA positivity was likely more frequent in the R1 resection group (86.7% vs. 58.9%; *p* = 0.105), while Synaptophysin staining remained consistently high in both groups (93.3% vs. 100%; *p* = 0.476).

**Figure 2:**
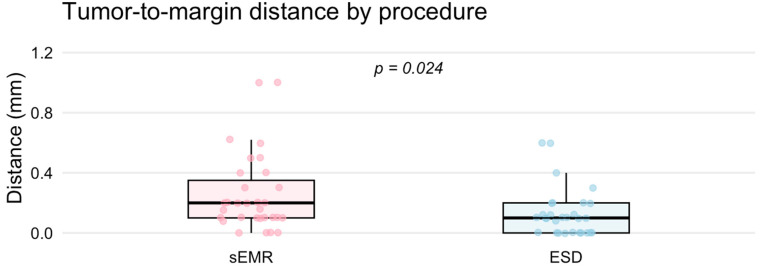
The tumor-to-margin distance was significantly greater in the sEMR group compared with the ESD group (median [IQR]: 0.2 [0.1–0.3] mm vs. 0.1 [0–0.2] mm, *p* = 0.024).

In addition to assessing R0 resection rate, we also measured the distance between the tumor and the resection margin under the microscope. This tumor-to-margin distance was significantly greater in the sEMR group compared with the ESD group [median (IQR): 0.2 (0.1–0.3) mm vs. 0.1 (0–0.2) mm; *p* = 0.024] (Figure 2), indicating a more generous histological margin achieved by sEMR.

### 3.5. Multivariate Analysis of Margin Status

To identify independent predictors of R1 resection, a Firth’s penalized logistic regression model was constructed (Table 4). Procedure type, lymphovascular invasion, and CgA positivity were included based on their clinical relevance and suggestive associations in univariate analysis.

Both ESD and lymphovascular invasion were independently associated with significantly increased odds of R1 resection. Patients who underwent sEMR had 0.19-fold odds of margin positivity compared to those who underwent ESD (95% CI: 0.03–0.72; *p* = 0.013). Lymphovascular invasion exhibited a particularly strong association, with an odds ratio of 28.7 (95% CI: 1.94–445; *p* = 0.012). Although CgA positivity was retained in the model, it did not reach statistical significance (OR: 3.03; 95% CI: 0.74–17.54; *p* = 0.130).

## 4. Discussion

Endoscopic resection is the recommended treatment for rectal NETs smaller than 10 mm [6], yet the optimal technique remains uncertain due to evolving endoscopic technologies and limited comparative data. Given that most rectal NETs extend into the submucosa or deeper layers [23,24], it was thought that simple polypectomy and conventional EMR often failed to achieve complete histological clearance and were therefore not considered first-line techniques. While endoscopic submucosal dissection (ESD) has been shown to be effective and safe for rectal NETs [17], its complexity and higher procedural demands have limited its broader application. To enhance the completeness of EMR, multiple modifications have been introduced to improve lesion suction and improve the R0 rate [11,12,13,14,15,16]. Furthermore, a recently proposed cap-assisted EMR technique that omits submucosal injection in selected patients [20] and another ESMR-L without submucosal injection [21] suggest that this traditionally essential step, submucosal injection, may be safely bypassed.

In this context, we conducted a retrospective cohort study comparing a simplified EMR (sEMR) technique—notably performed without submucosal injection—to conventional ESD. We found that sEMR achieved a higher complete resection rate and a lower bleeding risk, suggesting it may be a safer and more efficient option for selected patients with small rectal NETs.

The higher R0 resection rate observed in the sEMR group may be attributed, at least in part, to the technical features of the procedure, which is consistent with a significantly greater tumor-to-margin distance in the sEMR group. In our procedure, a clip was applied at the base of the lesion to lift it prior to resection. This maneuver likely facilitated sufficient tissue traction and enhanced lesion protrusion, leading to an adequate vertical resection depth. This wider histological clearance zone around the tumor may contribute to the higher likelihood of negative margins.

The omission of submucosal injection in the sEMR group may have contributed to the lower bleeding rate observed in our study. Although submucosal injection is typically used to elevate the lesion and protect deeper layers, it can also expand the submucosal space and increase the risk of vessel injury during dissection. In contrast, the simplified sEMR approach involves limited tissue manipulation, which may reduce vascular trauma and subsequent bleeding.

In addition to resection quality and safety, procedural efficiency is a critical consideration in clinical practice. In our study, the sEMR group demonstrated a significantly shorter operation time compared to the ESD group, likely due to the elimination of sophisticated submucosal dissection steps and the omission of submucosal injection. This streamlined workflow reduces not only the duration of the procedure but also operator fatigue and endoscopy suite utilization, potentially improving patient throughput. Furthermore, the material cost associated with sEMR was significantly lower than that of ESD. This difference is attributed to the reduced need for specialized equipment like electrosurgical knives and injection needles required for ESD. These findings highlight the procedural and economic advantages of sEMR, particularly in resource-limited settings or high-volume centers where efficiency and cost-effectiveness are essential.

Beyond the resection method, tumor-related factors were also analyzed for their association with margin status. While larger tumor size can reduce the complete resection rate, the similar tumor size distribution in both groups likely minimized its impact, and it was not found to be a significant risk factor in our analysis. Lymphovascular invasion (LVI) was significantly more frequent in patients with R1 resection, and Chromogranin A (CgA) positivity, though it did not reach statistical significance, was more common in this group. Nevertheless, both LVI [25] and CgA expression [26] may be indicative of more biologically aggressive tumors. These findings highlight that tumor biology—beyond the choice of resection method—may influence histologic outcomes.

To further examine whether the observed association between resection technique and the risk of R1 resection was confounded by tumor-related variables, we performed a Firth logistic regression analysis (Table 4). Even after adjusting for these factors, the resection method remained an independent predictor of margin status. Patients undergoing sEMR continued to exhibit a significantly lower risk of R1 resection compared to those treated with ESD, reinforcing the procedural advantage of this simplified technique.

Several limitations of this study should be acknowledged. First, the retrospective nature of the study design inherently introduces potential selection bias and limits the ability to establish causal relationships. Although we attempted to control for confounding variables through matching and multivariate analysis, unmeasured factors may still have influenced the outcomes. Second, this was a single-center study conducted at a tertiary academic hospital, which may limit the generalizability of our findings to other clinical settings with different endoscopic expertise or patient populations. Third, the sample size, while sufficient to detect significant differences in primary outcomes, may have been underpowered to assess more subtle associations, such as those involving immunohistochemical markers like CgA. Additionally, this study did not account for potential fluctuations in medical or procedural costs over time. However, given the substantial cost difference between the two techniques, this limitation is unlikely to alter the overall conclusion. Prospective, multicenter studies with larger cohorts are needed to validate our findings and refine patient selection criteria for simplified resection techniques.

In this exploratory study, the sEMR technique—performed without submucosal injection—showed a higher complete resection rate and lower bleeding risk compared to ESD for small rectal NETs, with shorter operation time and lower costs. These findings suggest that sEMR may be a safe and efficient alternative in selected patients.

## Figures and Tables

**Table 1:** Clinical characteristics and endoscopic procedure of patients.

	sEMR Group (*n* = 37)	ESD Group (*n* = 37)	*p* Value
Age	49.4 ± 11.0	48.6 ± 10.3	0.752
Male/Female	16/21	16/21	1.000
Clinical symptoms			0.878
Incidental finding	25 (67.6%)	28 (75.7%)	
Abdominal pain/distention	6 (16.2%)	4 (10.8%)	
Change in bowel habits	5 (13.5%)	4 (10.8%)	
Others	1 (2.7%)	1 (2.7%)	
Distance from anal verge (cm)	5 (5–8)	5 (4–8)	0.611
Tumor size (mm)	5 (5–7)	6 (4–8)	0.804
Endoscopy ultrasound before operation	31 (83.8%)	28 (75.7%)	0.563
En bloc resection	37 (100%)	37 (100%)	1.000
Intraoperative bleeding	1 (2.7%)	8 (21.6%)	0.028 *
Perforation	0	0	1.000
Operation time (second)	409 (324–648)	1469 (1106–1803)	<0.001
Material cost (CNY)	1486 (1341–1565)	6390 (5159–7713)	<0.001

* *p* < 0.05.

**Table 2:** Histological finding of different procedure group.

	sEMR Group (*n* = 37)	ESD Group (*n* = 37)	*p* Value
Tumor grade			0.560
G1	31	32	
G2	3	4	
Indeterminate	3	1	
Resection margin			0.019 *
R0 resection	34	25	
R1 resection	3	12	
Lymphovascular invasion	1 (2.7%)	2 (5.4%)	1.000
Immunohistochemistry for Chromogranin A	22/34 (64.7%)	24/36 (66.7%)	1.000
Immunohistochemistry for Synaptophysin	35/35 (100%)	35/36 (97.2%)	1.000

* *p* < 0.05.

**Table 3:** Univariate comparison of clinical and histological features between R1 resection and R0 resection groups beyond procedures.

	R1 Resection Group (*n* = 15)	R0 Resection Group (*n* = 59)	*p* Value
Age	49.0 ± 9.7	49.0 ± 10.9	0.996
Male/Female	6/9	26/33	1.000
Clinical symptoms			0.514
Incidental finding	12 (80.0%)	41 (69.5%)	
Abdominal pain/distention	1 (6.7%)	9 (15.3%)	
Change in bowel habits	1 (6.7%)	8 (13.6%)	
Others	1 (6.7%)	1 (1.7%)	
Distance from anal verge (cm)	7 (5–10)	5 (5–8)	0.229
Tumor size (mm)	6 (5–7.5)	5 (4–7.5)	0.362
Endoscopy ultrasound before operation	10 (66.7%)	49 (83.1%)	0.294
En bloc resection	15 (100%)	59 (100%)	1.000
Intraoperative bleeding	3 (20.0%)	6 (10.2%)	0.550
Perforation	0	0	1.000
Tumor grade			0.571
G1	14 (93.3%)	49 (83.1%)	
G2	1 (6.7%)	6 (10.2%)	
Indeterminate	0	4 (6.8%)	
Lymphovascular invasion	3 (20.0%)	0	0.006 *
Immunohistochemistry for Chromogranin A	13/15 (86.7%)	33/56 (58.9%)	0.105
Immunohistochemistry for Synaptophysin	14/15 (93.3%)	56/56 (100%)	0.476

* *p* < 0.05.

**Table 4:** Multivariate Firth logistic regression analysis for predictors of R1 resection.

Variable	Odds Ratio (OR)	95% Confidence Interval	*p* Value
Procedure (sEMR vs. ESD)	0.19	0.03–0.72	0.013 *
Lymphovascular invasion	28.7	1.94–445	0.012 *
Immunohistochemistry	3.03	0.74–17.54	0.130
for Chromogranin A			

* *p* < 0.05.

## Data Availability

The original contributions presented in the study are included in the article, further inquiries can be directed to the corresponding author.
